# Smart Partitioned Blockchain

**DOI:** 10.3390/s24134033

**Published:** 2024-06-21

**Authors:** Basem Assiri, Hani Alnami

**Affiliations:** Computer Science Department, Jazan University, Jazan 82917, Saudi Arabia; hmalnami@jazanu.edu.sa

**Keywords:** blockchain, partitioned blockchain, machine learning, deep learning, data sensitivity, security, smart spaces, Internet of Things

## Abstract

Blockchain is a developing technology that promises advancements when it is applied to other fields. Applying blockchain to other systems requires a customized blockchain model to satisfy the requirements of different application fields. One important area is to integrate blockchain with smart spaces and the Internet of Things to process, manage, and store data. Actually, smart spaces and Internet of Things systems include various types of transactions in terms of sensitivity. The sensitivity can be considered as correctness sensitivity, time sensitivity, and specialization sensitivity. Correctness sensitivity means that the systems should accept precise or approximated data in some cases, whereas time sensitivity means that there are time bounds for each type of transaction, and specialization sensitivity applies when some transactions are processed only by specialized people. Therefore, this work introduces the smart partitioned blockchain model, where we use machine learning and deep learning models to classify transactions into different pools according to their sensitivity levels. Then, each pool is mapped to a specific part of the smart partitioned blockchain model. The parts can be permissioned or permissionless. The permissioned parts can have different sub-parts if needed. Consequently, the smart partitioned blockchain can be customized to meet application-field requirements. In the experimental results, we use bank and medical datasets with a predefined sensitivity threshold for classification accuracy in each system. The bank transactions are critical, whereas the classification of the medical dataset is speculative and less critical. The Random Forest model is used for bank-dataset classification, and its accuracy reaches 100%, whereas Sequential Deep Learning is used for the medical dataset, which reaches 91%. This means that all bank transactions are correctly mapped to the corresponding parts of the blockchain, whereas accuracy is lower for the medical dataset. However, acceptability is determined based on the predefined sensitivity threshold.

## 1. Introduction

Blockchain is a well-designed technology that was initially developed for digital cryptocurrencies such as Bitcoin [1]. Blockchain technology has the ability to apply many concepts and values such as transparency, sharing, decentralization, and security. It uses distribution and decentralization approaches to replace trust, as trust is usually implemented through a centralized third party. Therefore, it has been proposed for use in many other areas, such as education, healthcare, and national security [2,3,4,5]. One important application is to integrate blockchain with smart spaces and the Internet of Things (IoT) to process, manage, and store data. Actually, smart spaces and IoT systems include various types of sensitivity transactions. However, the adoption of blockchain requires the modification and development of its architecture, algorithm, and protocols [6,7].

Indeed, blockchain consists of a peer-to-peer network that connects many nodes, where each node has a copy of a ledger. The ledger contains a chain of all approved blocks, where each block contains a group of valid transactions. The blockchain algorithm starts with transaction validation, where nodes validate transactions and group them into blocks. The nodes compete to push their blocks to the network for another level of validation. When a node succeeds in pushing a new block to the network, the other nodes validate the block to approve or disapprove it. Only the approved blocks are added to the ledger [8].

In fact, there are different types of blockchain, which are public, private, consortium, and hybrid. Firstly, the public blockchain is a permissionless system where anyone can join the network to process transactions, blocks, and ledgers. It is used in famous cryptocurrencies such as Bitcoin and Ethereum [9,10]. Secondly, a private blockchain is a permissioned system that restricts participation to specific members. Only those authenticated members can join the network and have the encryption keys to process transactions, blocks, and ledgers. This can be used internally by a company or an organization such as Corda and Quorum [10]. Thirdly, a consortium blockchain is a permissioned private blockchain that works across multiple organizations. It enables different companies and organizations to join the network. It can be used for interleaved businesses such as supply chains [9,10]. Fourthly, a hybrid blockchain is permissioned and permissionless, where the data and ledger are permissioned so that the network is private from one side. However, only some transactions are permissionless. Those transactions become available to the public for validation as needed [11]. Table 1 illustrates the main criteria of each type of blockchain.

In addition, smart spaces and IoT provide different kinds of transactions in which transaction is the main unit of blockchain data. Having different kinds of transactions requires different procedures. Therefore, at the beginning, the system’s transactions are classified according to their sensitivity into sensitive and non-sensitive transactions. The sensitive transactions need more privacy, authorization, or specialization. For example, in healthcare systems, some transactions need a specialized doctor to process them, so the public cannot process them. Another example is that some transactions need more privacy or security, such as those related to politics, business competition, and national security. Those transactions also cannot be revealed to the public, even with hidden identities. Now, this work facilitates the application of blockchain to different areas. However, the four mentioned types of blockchain each have their own vulnerabilities.

Therefore, this work presents a new type of blockchain, namely the smart partitioned blockchain. The partitioned blockchain has two parts: the first part is permissionless, and the other part is permissioned. The permissionless part is a part of the network that enables accessing, processing, and storage of non-sensitive transactions. In contrast, the permissioned part of the network enables accessing, processing, and storage of sensitive transactions. Moreover, the transaction classification can be predefined or smart. This work proposes a smart partitioned blockchain wherein another frontal layer is added to classify the transactions before they are introduced to the blockchain parts. The smart classification will be conducted using machine learning (ML) and deep learning (DL) models. The transactions are classified into many categories, such as sensitive, normal, or non-sensitive and then assigned to the proper blockchain parts.

The experimental results employ the Random Forest Classifier and a Sequential Deep Learning algorithm for practical implementation. The Random Forest model is applied to identify the status of bank transactions, and the classification accuracy reaches 100%, which means that each transaction is assigned to the appropriate part of the blockchain. The Sequential Deep Learning algorithm is utilized to predict medical-transaction statuses, and its classification accuracy reaches 91%. Both results are considered significant in terms of system sensitivity requirements, which will be explained in detail in Section 4, Section 5 and Section 6.

The paper’s remainder is organized as follows: Section 2 shows the related work; Section 3 demonstrates the methodology, and Section 4 shows the dataset and preprocessing details. Section 5 discusses modeling and evaluation, whereas Section 6 discusses experimental results. Finally, Section 7 concludes the paper.

## 2. Related Work

The blockchain types and algorithms are discussed in many works. Yang et al. study private and public blockchain models [9], whereas the hybrid blockchain is investigated in another work [11]. Krishnan et al. introduces a comprehensive study of all types [10].

Recently, many works have modified blockchain algorithms to overcome the limitations and address the needs of applications in different areas. Some researchers modify the blockchain general models or algorithms and involve the concept of leader election [12,13]. Liyanaarachchi et al. focus on socio-technical decision-making in the blockchain, so they imply selective immutability and federal decentralization concepts [14]. Some others relax the blockchain models to reduce the cost or to speed up the processing time. This helps to apply blockchain technology in many other fields that cannot handle processing costs and delays. For example, a blockchain model has been relaxed to enhance cross-border data sharing in [15].

In addition, many consensus protocols have been introduced to give the mining process more flexibility and to avoid limitations. The typical consensus protocol is Proof-of-Work (PoW), where the mining process includes solving a complicated mathematical puzzle [16]. PoW provides more security and decentralization, but it suffers from being time and energy consuming. After that, Proof-of-Stake (PoS) and Delegated Proof-of-Stake (DPoS) are introduced to reduce the processing cost and energy consumption of PoW [16,17]. In PoS and DPoS, the miner is selected based on the amount of cryptocurrency it has. However, in PoS and DPoS, the rich nodes can control the system processes. Additionally, Proof-of-Authority is another consensus protocol that relies on authorized validators who meet the transaction requirements, but it also affects blockchain decentralization [18]. One of the famous consensus protocols is the Practical Byzantine Fault Tolerance (PBFT), which tolerates partial errors and approves majority decisions [17,18]. However, such tolerance is not acceptable for critical systems. This variety among consensus protocols enables support provision for different systems.

Moreover, the main consideration of the four types of blockchain is security. The public blockchain uses decentralization in the processing and distribution of storage (ledger). Meanwhile, the private blockchain provides higher security as it relies on permissioned access (access restriction). The consortium blockchain also relies on permissioned access to support security, but it is expanded to work across organizations rather than being private and restricted to one organization. The hybrid blockchain combines permissioned and permissionless techniques as it generally works as a private blockchain but only introduces part of transactions to the public for validation [19]. The hybrid blockchain is not open, which highlights the need for another type, namely that is the smart partitioned blockchain that has a specific partition that enables the public to not only validate transactions, but can also share data, validate blocks, vote, and access the ledger. Indeed, the smart partitioned blockchain comprises different parts, with some being fully permissioned and others fully permissionless. Further details are provided in Section 3. 

Furthermore, systems address the time costs of blockchain algorithms. Some systems enable scalability solutions by validating transactions off-chain or in sidechains [20,21,22], which reduces the contention of the blockchain network, as is done in Bitcoin and Ethereum [23,24]. On the other hand, supporting blockchain with other innovative technologies, such as zero-knowledge proofs and efficient encryption techniques, is a vital step [25]. However, this work uses ML and DL models for more efficiency and automation. Actually, ML and DL models enhance efficiency by improving productivity and scalability. They also enhance automation by reducing human involvement and biases. Additionally, the ML and DL models help to find sensitive cases so that they are treated carefully by directing them to the suitable part of the blockchain to maintain the required safety and security.

## 3. Methodology and Architecture

**Figure 1:**
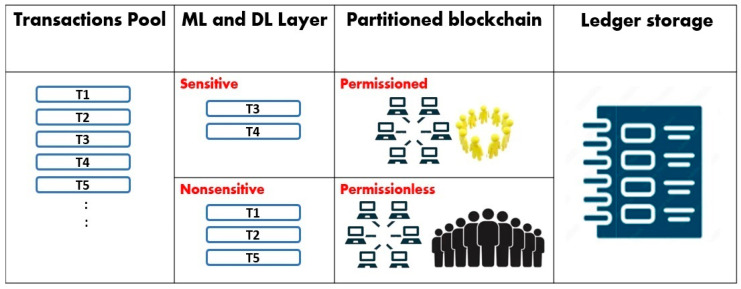
Shows a smart partitioned blockchain with two categories of transactions, which are sensitive and non-sensitive. Consequently, the partitioned blockchain layer has a permissioned part and a permissionless part. The last layer shows the ledger storage.

At the beginning, transactions are generated by users and are put in the public transactions pool. Figure 1 shows the general architecture of the smart partitioned blockchain, which has three major phases. Upon receiving transactions from IoT sources, the first phase is for transaction classification, the second phase is to map the transaction classes to the partitioned blockchain, whereas the third phase focuses on ledger storage management.

Firstly, transactions generated from IoT sources are grouped in a transaction pool. The ML and DL models are used to classify transactions. The classes are determined based on the sensitivity of transactions. In fact, some systems can use ML models, and others use DL models according to the accuracy of the transaction-classification process. For each system, many models are tested, and the model with the highest accuracy is selected. This flexibility supports the ability to classify different kinds and different structures of transactions. The transactions’ types and classes can be predefined. However, the application of ML and DL in the classification process enhances automation and reduces biased human-factor involvement. In addition, after training the ML and DL models, the rules and classification patterns can be transformed into smart contracts. This helps to speed up the classification process.

**Figure 2:**
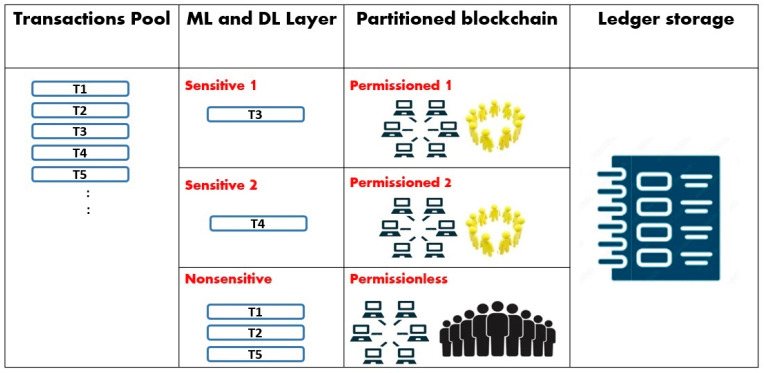
Shows a smart partitioned blockchain with three categories of transactions. The partitioned blockchain layer has two sub-parts of permissioned blockchain and one permissionless blockchain part. The last layer shows the ledger.

Secondly, as the blockchain is partitioned, each class of transactions is mapped to the suitable part of the blockchain. Indeed, the blockchain is partitioned according to the system’s needs. For example, if a system requires different security levels, then the sensitive transactions are processed using the permissioned part of the blockchain, and the non-sensitive transactions are processed using the permissionless part of the blockchain. Another example is that if a system requires different levels of specialization, then the normal transactions are mapped to the permissionless part of the blockchain, whereas the sensitive transactions that require specialization are processed using the permissioned part of the blockchain. If there are different kinds of specializations, then the permissioned part of the blockchain is divided into many sub-parts, and the transactions are classified accordingly, as shown in Figure 2.

Thirdly, the ledger-management process manages data availability and accessibility. As there are different levels of data sensitivity, data-access control is required. Thus, for ledger-access-control management, we suggest three different mechanisms that suit different kinds of systems, which are explained as follows:

- Separate ledgers: If the transaction classes are fully independent, then each part of the blockchain stores the approved blocks in its own ledger. This means that each part has a separate ledger.
- The general ledger: All approved blocks are stored in the same ledger regardless of which part generates them.
- Hierarchical ledger: In this case, the system has two ledgers—local and general. The permissionless part has its own ledger (the local ledger), so any block approved by the permissionless part is stored in the local ledger, and another copy of each block is stored in the general ledger as well. On the other hand, all approved blocks of the permissioned part are stored in the general ledger. Therefore, the general ledger includes all approved blocks within the system (from all parts), whereas the local ledger only shows the blocks of the permissionless part. This helps to separate public data from private data.

## 4. Dataset Description and Preprocessing

### 4.1. Dataset Description

This study analyzed two distinct datasets to investigate different kinds of smart spaces, which are bank and medical data. The bank-transaction dataset comprises a detailed record of transactions, ATM locations, transaction types, cardholder information, and temporal data from Wisabi Bank [26]. Key features within the bank dataset are outlined in Table 2, with the primary aim being to identify transaction status, which is categorized into three types: sensitive, non-sensitive, and soft-sensitive transactions (soft-sensitive is a category that lies between sensitive and non-sensitive). The target variable is derived by associating values from the TransactionType feature, encompassing withdrawals, deposits, balance inquiries, and transfer transactions. Withdrawals and transfers are classified as sensitive due to their direct influences on the available balance, whereas deposits are considered soft-sensitive and balance inquiries are non-sensitive. The dataset contains a total of 2,143,838 transactions.

The medical-transaction dataset is sourced from the Centers for Disease Control and Prevention (CDC) and encompasses various data on heart disease and its associated factors [27]. The CDC accumulated over 100,000 medical records in 2020, containing several crucial and sensitive variables such as BMI, smoking habits, physical activity levels, and general health indicators, as detailed in Table 3. According to CDC findings, half of the American population exhibits at least one of three major risk factors for heart disease, namely smoking, high cholesterol, and high blood pressure. This study endeavors to differentiate between sensitive and non-sensitive medical records using a binary classification deep learning algorithm in order to reduce the prevalence of heart disease by facilitating early medical interventions.

### 4.2. Data Preprocessing

The dataset concerning bank transactions was employed to develop a detection model to identify transactions as sensitive, non-sensitive, or soft-sensitive. Consequently, transaction types closely link the target variable, encompassing withdrawals, deposits, transfers, and balance inquiries. We categorized transaction types as follows: withdrawals and transfers were classified as sensitive transactions, deposits were classified as soft-sensitive, and balance inquiries were classified as non-sensitive.

**Figure 3:**
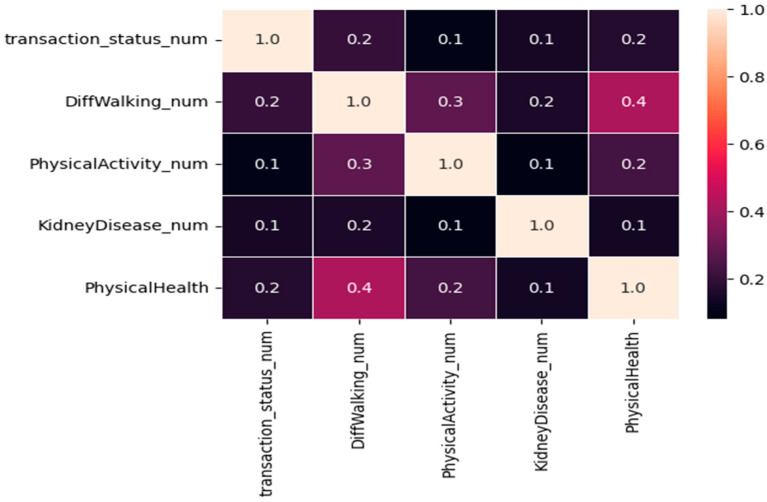
Correlation matrix of the health data; the target variable is 0 or 1, where 0 denotes a non-sensitive medical transaction, and 1 indicates a sensitive medical transaction. We classified transactions of patients experiencing a heart attack as sensitive transactions.

In the medical dataset, our approach began with converting categorical values into numeric equivalents using the pandas factorize function. We subsequently applied correlation feature selection to identify variables that were strongly associated with our target variable, as depicted in Figure 3. According to the correlation heat map, variables such as physical health and activity, difficulty climbing stairs, and kidney disease exhibited robust and positive correlations with the target variable representing transaction status. 

## 5. Modeling and Evaluation

In this study, we employed a Random Forest Classifier and a Sequential Deep Learning algorithm for practical implementation. The Random Forest model was applied to identify the status of bank transactions, whereas the Sequential Deep Learning model was utilized for predicting medical-transaction statuses. Both models are recognized for their effective performance: Random Forest operates as an ensemble learning algorithm by amalgamating numerous decision tree models [28]. DL demonstrates proficiency in discerning intricate data patterns and relationships [29].

A Random Forest algorithm was constructed using 100 estimators and trained on 75% of the dataset to detect bank-transaction statuses. We split the bank dataset, containing over 2 million records, into training and testing sets using the train_test_split function available in the sklearn model_selection module [30]. Then, the transaction type ID was selected as the input feature, while the transaction status feature was the target. The algorithm identified withdrawal and transfer transactions as sensitive, classified deposit transactions as soft-sensitive, and categorized all balance-inquiry transactions as non-sensitive. To assess the efficacy of the Random Forest algorithms, we employed widely recognized evaluation metrics, including the confusion matrix, precision, recall, F1-score, and accuracy. We identified sensitivity thresholds by measuring how accurately the model identified actual positives for the following three classes: sensitive, soft-sensitive, and non-sensitive transactions. We used metrics such as recall and Receiver Operating Characteristic (ROC) curves to find the best threshold value for the Random Forest model.

In the context of the medical dataset, we employed a neural network architecture utilizing Keras, an interface facilitating the development of high-level neural networks atop TensorFlow. Specifically, we crafted a sequential model comprising an input layer, two hidden layers, and an output layer. The input layer was configured to receive input vectors (Difficulty climbing stairs, physical activity, kidney disease, and physical health) of four dimensions, while the output layer employs softmax activation to generate probability distributions for two classes (sensitive: has heart disease and non-sensitive: does not have heart disease). The structure of the hidden layers was delineated as follows: the initial hidden layer was composed of 64 neurons, employing a rectified linear activation function, and the subsequent layer consisted of 32 units, also employing the rectified linear activation function. Our model was configured with the Adam optimizer algorithm, which is a widely adopted method for optimizing neural network training that dynamically adjusts the learning rate during the training process. Accuracy, precision, recall, and area-under-the-curve metrics were employed throughout training to assess the model’s predictive performance relative to the ground truth labels and to determine the optimal sensitive-threshold value for the model. Training was conducted utilizing 80% of the available data for 16 epochs.

On the other hand, to apply smart partitioned blockchain on different domains, we should investigate the required sensitivity level of each system. Accordingly, we determined a threshold (A) to represent the minimum classification accuracy that can be accepted. For each system, A is predefined by the system admins according to the sensitivity level. For some systems, A is determined by international and national organizations through their standards, rules and regulations. For example, the World Health Organization, along with national food and drug administrations, define some numerical thresholds for critical products and processes. The World Trade Organization defines some numerical thresholds and standards for more products and processes. In additions, some systems should investigate their products and processes to find the sensitivity levels. For example, some systems rely on stakeholders’ feedback, revenue, and other statistics to measure their performance and adjust A over the time.

Indeed, each system has its sensitivity requirements. For example, some healthcare transactions, such as reading or updating patients’ health records, are very critical. Therefore, 100% accuracy is required (A = 100). Some other systems, such as speculation, prediction, decision support, and inventory systems, are less sensitive, and so the A can be between 85 and 95. Some systems, such as recommendation systems for restaurants or movies, are not sensitive [31,32,33].

This shows how critical it is to classify transactions in the right pools, and to be processed by the suitable part of the smart partitioned blockchain. Thus, ML and DL classification accuracy cannot be less than system A.

In our experiment, we used two datasets, namely the bank and medical datasets. The bank dataset is very sensitive, with A = 100. Within the bank dataset, there are different kinds of transactions, which are classified according to their sensitivity and then mapped to the right parts of the blockchain. Meanwhile, the medical dataset has some health records, including personal health records. The learning model should classify those records into sensitive and non-sensitive records in relation to heart disease. This is a speculative study to find those people who are more likely to be exposed to heart disease. In addition, due to speculation and the large number of risk factors for developing heart disease, the sensitivity of classification accuracy can be less than 100%, let us say A = 90, for example.

## 6. Results and Discussion

### 6.1. ML and DL Results

In our experiment, we used the Random Forest model as ML model, and Sequential Deep Learning as DL model. Those models are selected as examples to show how the transactions’ classification is applied as a layer in the smart partitioned blockchain. If the classification accuracy is less than the system threshold, then other models can be used instead.

**Figure 4:**
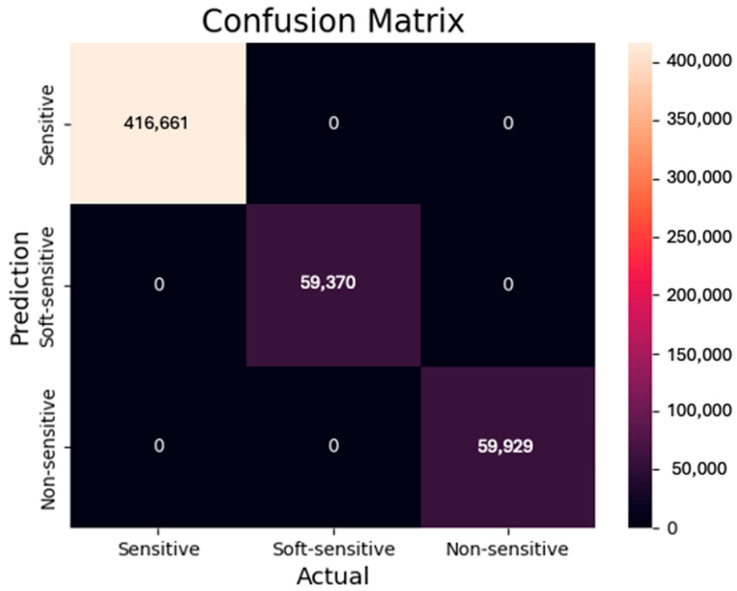
Confusion matrix of bank-transaction-status detection.

The Random Forest model exhibited a remarkable ability to detect bank-transaction statuses, as evidenced by its 100% accuracy in all evaluation metrics (Precision, recall, F1-score, accuracy, macro average, and weighted average) for all three classes. Additionally, the ROC curve indicates that the true positive rate of the ML model achieved optimal results, with a value of 1 or (100%) for all predicted classes. This result was achieved because the input variable (Transaction types) creates the target variable. In other words, the model links the input to the output to provide the final detection of transaction statuses, making it straightforward for the model to determine the transaction statuses based on the transaction type input feature. Figure 4 shows the confusion matrix of the Random Forest model.

The Sequential Deep Learning algorithm demonstrated exceptional performance across all evaluation metrics. Table 4 summarizes the model’s performance: 91% accuracy, precision, and recall, with an AUC of 92%. The accuracy of medical transactions classification is slightly lower than that of the bank transactions. This is because the target variable has no direct link with the input features, unlike in the case of the bank transactions. The deep learning model aims to predict whether a person has heart disease based on difficulty climbing stairs, physical activity, kidney disease, and physical health. Using only these features, the deep learning model successfully predicts the medical condition with over 90% accuracy across all evaluation metrics.

### 6.2. Smart Partitioned Blockchain

As mentioned earlier, we should determine the sensitivity level of the smart space and IoT systems in general. Then, we determine the type of transactions, the quantity of each type, and the sensitivity level of each type. Consequently, we decide how many parts should be in the smart partitioned blockchain and the scalability of each part. For example, if the system has three types of transactions, and one of them is very sensitive, whereas the other two types are not sensitive, then the smart partitioned blockchain should have two parts, with one part permissioned for the very sensitive transactions, and a permissionless part to which the other two types are mapped.

**Figure 5:**
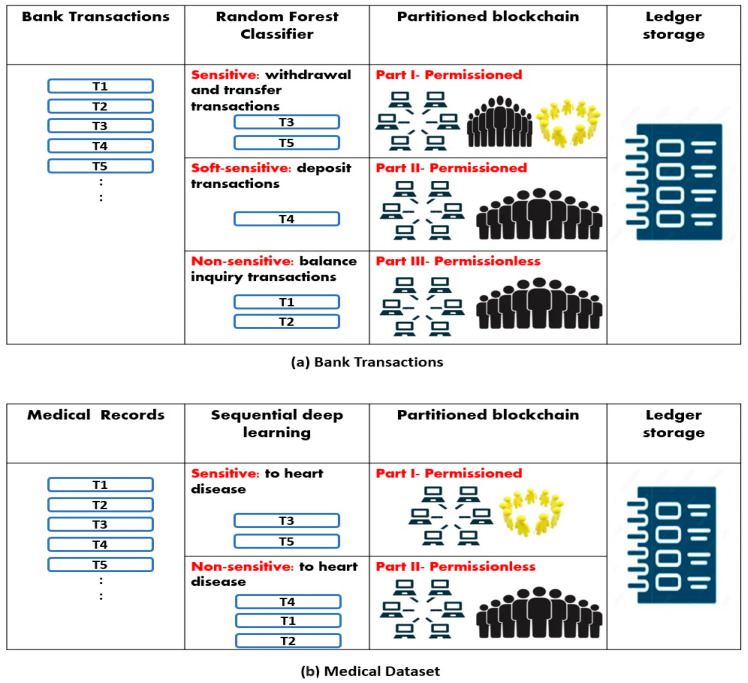
Part (**a**) shows the application of smart partitioned blockchain on the bank dataset with three categories of transactions that are mapped to three parts of the blockchain, whereas part (**b**) shows the application of a smart partitioned blockchain to the medical dataset, with two categories that are mapped to two parts of the blockchain.

As shown in Figure 5a, our experiment uses the bank dataset, which has four kinds of transactions. Every kind has a different structure, which makes it easy for them to be recognized. The kinds of transactions are withdrawals, transfers, deposits, and balance-inquiry transactions. The ML classifies withdrawals and transfers transactions as sensitive, and they are mapped to part I of the smart partition blockchain (permissioned). Deposit transactions are classified as soft-sensitive, and they are mapped to part II of the smart partition blockchain (permissioned). The balance-inquiry transactions are classified as non-sensitive, so they are mapped to part III of the smart partition blockchain (permissionless).

The system sensitivity can be defined as correctness sensitivity, time sensitivity, or specialization sensitivity. The bank system’s sensitivity is considered to include correctness sensitivity and time sensitivity. Therefore, all transactions have a bounded time to execute, and the sensitive transaction must be executed immediately and correctly. The time is relaxed a little for the soft-sensitive transactions, but it must be correct. For non-sensitive ones, the correctness is relaxed. Indeed, time relaxation results in correctness relaxation. For example, if an account x contains USD1000 and someone deposits USD100, and concurrently someone issues a balance-inquiry transaction, then due to the relaxation of the deposit transaction as it is soft-sensitive, the balance inquiry will return USD1000, and the new deposited value will not appear.

As shown in Figure 5b, our experiment also uses the medical dataset, which contains personal health records. The records are classified as sensitive or non-sensitive. The sensitive transactions are mapped to part I of the smart partition blockchain (permissioned), whereas the non-sensitive transactions are mapped to part II of the smart partition blockchain (permissionless). The personal health records that are classified as sensitive are mapped to the permissioned part that has specialized doctors. The non-sensitive transactions are mapped to the permissionless part that has general doctors.

Finally, as mentioned earlier, the bank dataset is very sensitive where A = 100, and the accuracy of the classification process of the ML model is 100%. On the other hand, the medical system applies a speculative study, and thus the sensitivity of classification accuracy can be less than 100% and the DL model reaches 91% accuracy.

### 6.3. Discussion

The smart partitioned blockchain has many potential advantages. Firstly, the smart partitioned blockchain out performs the other four types of blockchain. It out performs permissioned and permissionless blockchain types as it combines both of them to obtain the advantages of both. It also out performs the consortium type as it is an expanded permissioned blockchain that runs across organizations, whereas the smart partitioned blockchain includes a permissionless part that provides more scalability, productivity, and decentralization. It out performs the hybrid blockchain type that works as a private blockchain but only introduces part of transactions to the public for validation. The smart partitioned blockchain has a specific part that enables the public to not only validate transactions, but to also share data, validate blocks, vote, and access the ledger. The smart partitioned blockchain has the advantage of allowing for the processing and storage of both sensitive and non-sensitive transactions in a single network. This can provide better privacy and security for sensitive transactions while still allowing for transparency and decentralization. Secondly, the smart partitioned blockchain supports blockchain adaption in different areas through the ability of create customization. The customization is built on the nature of data, processing requirements, network capacity, and storage requirements. Thirdly, the smart partitioned blockchain integrates blockchain with ML and DL, for more efficiency and automation. Actually, ML and DL models enhance efficiency by improving productivity and scalability. They also enhance automation by reducing human involvement and biases. Additionally, the ML and DL models help to find sensitive cases so that they are treated carefully by directing them to the suitable part of the blockchain to maintain safety and security.

On the other hand, there are many challenges that may result in some disadvantages when applying the smart partitioned blockchain in different areas. Firstly, blockchain processes data in a transactional form; however, transforming the data into a transactional form is critical process. For example, in IoT, part of the data comes from sensors in the form of signals, and transforming them into a transactional form is critical. Secondly, determining the block structure, size, content, and hashing techniques for each domain is difficult. Thirdly, for each system, it is hard to define the suitable network size, communication protocol, transmission security, and encryption standards. Fourthly, selecting the suitable ML and DL models for each system requires deep theoretical and experimental investigations. Fifthly, finding the proper accuracy thresholds is another critical point that was already discussed in Section 5. Sixthly, selecting the proper ledger type (among those presented) is another issue. In fact, further research is required to overcome such challenges, although researchers can rely on smart partitioned blockchain as a customizable model that facilitates application to other domains.

## 7. Conclusions

To integrate blockchain with other smart spaces and IoT, this work introduces a smart partitioned blockchain model that uses ML and DL models to classify transactions into different pools according to their sensitivity levels. Then, each pool is mapped to a specific part of the smart partitioned blockchain model. The parts can be permissioned or permissionless. The permissioned parts can have different sub-parts if needed. The experiments show that the Random Forest model is used for bank-dataset classification and that its accuracy reaches 100%, whereas Sequential Deep Learning is used for the medical dataset and its accuracy reaches 91%. The acceptability of the degree of accuracy is decided according to the predefined sensitivity threshold for each system. In the future, we suggest using different ML and DL models to enhance the classification accuracy. In addition, we are working on applying a smart partitioned blockchain to a specific domain to maintain all requirements using smart contracts and consensus protocols.

## Figures and Tables

**Table 1:** Types of Blockchain.

Criteria	Public	Private	Consortium	Hybrid
**Network**	Permissionless	Permissioned	Permissioned	Permissioned network but some transactions are permissionless
**Consensus**	All members	Organization members	Authorized members across organizations	Authorized members
**Decentralization**	Very High	Low	Low	High
**Security**	High, because of decentralization and ledger distribution	Very High, because of permissioned access, authentication, and encryption properties	Very High, because of permissioned access, authentication and encryption properties	Very High, because it acts as a private blockchain
**Threat**	The openness can introduce the risk of 51% attacks, denial of service attacks, and sybil attacks	Centralization can lead to a specific group controlling the blockchain decisions	Cannot guarantee trust among organizations that have different interests	Transparency issues can affect the output, as public transactions have hidden data
**Scalability**	Very High	Low	High	High
**Limitations**	- Lack of authentication - Lack of customization	- Centralization - Lack of scalability - Lack of customization	- Centralization - Lack of scalability - Lack of customization	- Partial Centralization - Partial scalability - Lack of customization - Lack of transparency as data is shielded

**Table 2:** Wasabi Bank Variable Descriptions.

Variables	Description
**Transaction ID**	Represent the ID of each transaction
**Transaction Start and End Time**	Represent the start and end time of the transaction in the format (Year/Month/Day), Hour: Minutes
**Cardholder ID**	The ID of the cardholder
**Location ID**	Location ID of where the transaction is made
**Transaction Amount**	Amount of transaction
**Transaction Type ID**	The types of the transactions consist of withdrawal, deposit, balance-inquiry, and transfer transactions
**Transaction Status (Target)**	Is derived by associating values from the transaction type where withdrawal and transfer are classified as sensitive, whereas deposit and balance inquiries are classified as soft-sensitive and non-sensitive, respectively

**Table 3:** Heart Disease Dataset Variable Descriptions.

Variables	Description
**Heart Disease**	Did the patient have heart disease? (Yes or No)
**BMI**	Body Mass Index measurement
**Smoking**	Does the patient have smoking habits? (Yes or No)
**Alcohol Drinking**	Does the patient drink alcohol? (Yes or No)
**Stroke**	Did the patient have a stroke? (Yes or No)
**Physical Health**	How many days a month did the patient feel poor physical health?
**Mental Health**	How many days a month did the patient feel poor mental health?
**Difficulty Walking**	Difficulty climbing stairs
**Diabetic**	Does the patient have diabetes? (Yes or No)
**Physical Activity**	A patient who reported their physical activity in the past 30 days
**Gen Health**	General health (Fair, Good, Very Good, or Excellent)
**Sleep Time**	Number of hours of sleep
**Asthma**	Does the patient have asthma disease? (Yes or No)
**Kidney Disease**	Does the patient have kidney disease? (Yes or No)
**Skin Cancer**	Does the patient have skin cancer disease? (Yes or No)

**Table 4:** Evaluation Metrics Results of The Sequential Deep Learning Model.

Algorithm	Accuracy	Precision	Recall	AUC
Sequential	91%	91%	91%	92%

## Data Availability

The used datasets are cited. and the classification codes are openly available and are submitted as Supplementary Materials.

## Supplementary Materials

The following supporting information can be downloaded at: https://www.mdpi.com/article/10.3390/s24134033/s1, HealthCare Transaction Dataset-deep learning model_code.ipynb, experiment1; Wisabi Bank transaction Dataset-code.ipynb, experiment2.
